# Contrasting patterns of *RUNX*2 repeat variations are associated with palate shape in phyllostomid bats and New World primates

**DOI:** 10.1038/s41598-018-26225-7

**Published:** 2018-05-18

**Authors:** Tiago Ferraz, Daniela M. Rossoni, Sérgio L. Althoff, Alcides Pissinatti, Vanessa R. Paixão-Cortês, Maria Cátira Bortolini, Rolando González-José, Gabriel Marroig, Francisco M. Salzano, Gislene L. Gonçalves, Tábita Hünemeier

**Affiliations:** 1 Department of Genetics, Biosciences Institute, Federal University of Rio Grande do Sul, 91501-970 Porto Alegre, RS Brazil; 2 Department of Genetics and Evolutionary Biology, Biosciences Institute, University of São Paulo, 05508-900 São Paulo, SP Brazil; 3 Regional University of Blumenau, Blumenau, SC Brazil; 4 Biology Department, Federal University of Bahia, Salvador, BA Brazil; 5 Rio de Janeiro Primatology Center, 20940-200 Rio de Janeiro, RJ Brazil; 6 Patagonian Institute of Social and Human Sciences, National Council for Scientific and Technological Research-CONICET, U9120ACD Puerto Madryn, Argentina; 7 Department of Environmental Resources, Faculty of Agrarian Sciences, University of Tarapacá, Arica, Chile

## Abstract

Establishing the genetic basis that underlies craniofacial variability in natural populations is one of the main topics of evolutionary and developmental studies. One of the genes associated with mammal craniofacial variability is *RUNX*2, and in the present study we investigated the association between craniofacial length and width and RUNX2 across New World bats (Phyllostomidae) and primates (Catarrhini and Platyrrhini). Our results showed contrasting patterns of association between the glutamate/alanine ratios (Q/A ratio) and palate shape in these highly diverse groups. In phyllostomid bats, we found an association between shorter/broader faces and increase of the Q/A ratio. In New World monkeys (NWM) there was a positive correlation of increasing Q/A ratios to more elongated faces. Our findings reinforced the role of the Q/A ratio as a flexible genetic mechanism that would rapidly change the time of skull ossification throughout development. However, we propose a scenario in which the influence of this genetic adjustment system is indirect. The Q/A ratio would not lead to a specific phenotype, but throughout the history of a lineage, would act along with evolutionary constraints, as well as other genes, as a facilitator for adaptive morphological changes.

## Introduction

An understanding of the genetic basis associated with craniofacial variability in animal natural populations is a major focus of modern evolutionary and developmental studies^1–5^. Until recently, broad phenotypic differences were thought to be the result of several gene mutations, each one with relatively small effects^6^. This proposal was based on extrapolations from studies with housekeeping genes in model species, as well as on the high capacity of population genetics to predict the results of allelic frequency changes in natural populations^7^. However, through the use of large-scale DNA sequencing, current studies of genes that either regulate the development process or code for receptors and signalling complex metabolic networks indicate that some mutations can have a large effect on morphology^2,8,9^.

One of these genes, *RUNX*2 (Runt-related transcription factor 2), was suggested to have a large effect on mammals’ morphological variation, specifically on the craniofacial variability^10–12^. RUNX2 is one of the most important transcription factors associated with bone tissue formation, and is responsible for promoting cell transitions among cell lineages^13,14^ being essential for osteogenesis^15,16^. The *RUNX* family is composed by three genes that share the same DNA binding motif (Runt Domain): *RUNX1*, *RUNX2*, and *RUNX3*. However, *RUNX2* is the only one that contains a tandem amino acid repeat region that precedes this domain^17^. The indicated repeat motif presents one segment rich in glutamate residues (polyQ), followed by a segment rich in alanine (polyA)^10^. This region appears to play an important role in the repression and activation of essential proteins for craniofacial development^18^. Clinical genetic studies have demonstrated that haplo-insufficiency of the *RUNX2* gene is associated with cleidocranial dysplasia (CCD) in humans, an autosomal dominant disease characterized by systemic skeletal anomalies, causing shortening of the face, abnormalities in the permanent dentition, as well as stature effects in affected individuals^19–24^.

Fondon and Gardner^25^ evaluated the correlation between different amino acid repeat motifs present in developmental genes and their effects across dog breeds with diverse skeletal and craniofacial phenotypes. They found a positive correlation between the ratio of polyglutamines (Q) to polyalanines (A) in the Q/A repeat domain of the *RUNX2* gene and craniofacial length. Similar patterns of significant positive correlation between the *RUNX2* repeat and facial length was also observed in other mammals belonging to the order Carnivora^10^. In the same study, based on functional tests, Sears *et al*.^10^ found significant correlations between the ratios of polyglutamines to polyalanines in the *RUNX2* tandem repeat region and *RUNX2* transcriptional activity. Other studies also provided evidence for the polyQ and polyA regions as being a mechanism of fine tuning in the activation and repression of the RUNX2 protein^10,16^. Conversely, Pointer *et al*.^11^ found no correlation between *RUNX2* sequence and face length across a variety of non-carnivoran placental mammals. Also, recently, Newton *et al*.^12^ demonstrated that RUNX2 does not drive craniofacial diversity in marsupials. Taken together, these previous studies suggest that Q/A repeat domain of RUNX2 might play a role in craniofacial evolution in some mammalian lineages but not others. Therefore, the role of the Q/A repeat domain of RUNX2 in craniofacial evolution lineage must be investigated independently for each lineage.

In the present study we combine phylogenetic comparative methods and morphological integration analysis to investigate the association between craniofacial length and width with RUNX2 Q/A ratios across two mammalian lineages displaying large variation in skeletal terms, the New World leaf-nosed bats (Family Phyllostomidae) and Primates (Catarrhini and Platyrhini). The investigation of these taxa is of interest to understand the developmental mechanism involved in the expression of *RUNX2*.

**Figure 1:**
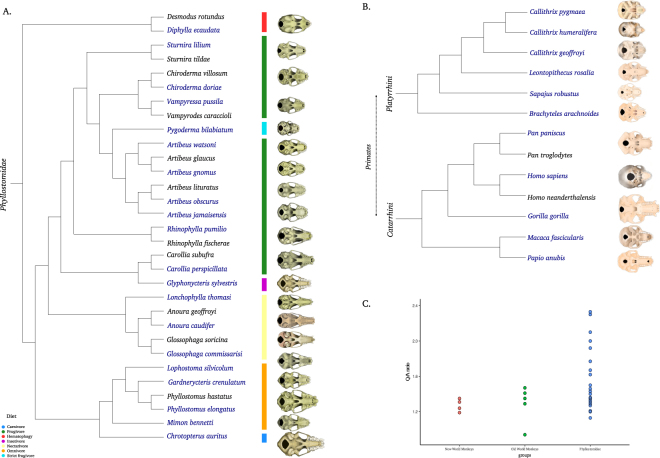
Phylogenies of sampled phyllostomid bats (**A**) and primates (**B**) (Old World and New World monkeys) showing a wide range of craniofacial diversity; (**C**) distribution of Q/A ratios for each group analysed. Species names in blue indicate the skull represented in the figure.

The New World leaf-nosed bats, Family Phyllostomidae, is a Neotropical lineage that evolved during the last 30 million years^26^ and represents one of the most ecologically diverse families of mammals^27^. They present a marked diversity in feeding specialisations, including species that consume insects, blood, small vertebrates, fruits, nectar and pollen. Feeding specialisation is accompanied by morphological adaptations, such as conspicuous craniofacial variability (Fig. 1A)^27–29^. Few studies have explored the genetic basis underlying diversity in craniofacial phenotype in bats. One of the rare studies to consider this subject was conducted by Phillips *et al*.^30^, who examined the evolutionary dynamics of the Pax9 transcription factor and craniofacial diversification in bats. Subsequently, Ball *et al*.^16^ found an association between the *RUNX2* variation and cranial bone density in bats and other mammals. On the other hand, the craniofacial morphological variation across phyllostomid lineages has been widely documented through biomechanical, functional, and developmental studies^27,29,31–42^.

From a comparative perspective, primates show remarkable morphological variation (Fig. 1B). The Catarrhini evolved during the last 35 million years^43^ and represents a monophyletic lineage encompassing all Old World monkeys (OWM) and apes, including humans. Catarrhines exhibit a diverse range of life histories, habitat use, and dietary strategies, and their geographic distribution includes tropical ecosystems in Africa and Asia^44^. The evolutionary diversification of the Platyrrhini (NWM) occurred over a period of more than 30 million years, driven by diet and size-related selection^45,46^. Species from both Catarrhini and Platyrrhini display diverse cranial morphologies and remarkable variation in body size. Among the studies with hominids, Green *et al*.^47^ hypothesized that an evolutionary change in RUNX2 affected the upper body and cranial morphology of modern humans. Additionally, as demonstrated by Lindskog *et al*.^48^, humans and macaques showed differences in protein and RNA expression levels in the *RUNX2* functional network genes (e.g. *RUNX1*, *SMAD3*, *SOX6*, and *SATB*). Other genomic studies showed a correlation between *RUNX2* and nose bridge breadth in current human populations^49^. However, in several other primate lineages, such as the NWM, little is known about the genetic basis that controls the fine-tuning of craniofacial phenotypic variation.

## Results

### Morphological variation and *RUNX2* repeat variability

Craniofacial variation is a conspicuous trait of phyllostomid bats. We observed marked differences in palate shape across species representing all feeding strategies (electronic supplementary material, Table S1 and Fig. 1A,B). The nectarivorous species (*Anoura caudifer* and *A. geoffroyi*; *Glossophaga commissarisi* and *G. soricina*; *Lonchophylla thomasi*) presented the longest (0.83–0.90 length/GM) and narrower face (0.51–0.54 cm width/GM), what is expected as a morphological adaptation to a diet based on nectar. Alternatively, the hematophagous (*Desmodus rotundus* and *Diphylla ecaudata*) showed the shortest (0.44–0.51 length/GM), but broader (0.60–0.62 width/GM) face, revealing a more robust cranium. *Glyphonycteris sylvestris*, an insectivorous species, also presented a long face (0.81 length/GM, 0.64 width/GM). Interestingly, frugivorous species presented the greatest variation of palate shape (0.60 length/GM, 0.78 width/GM, *Artibeus gnomus*, and 0.74 length/GM, 0.80 width/GM, *Chiroderma villosum*). The strictly frugivorous *Pygoderma bilabiatum* presented the shortest (0.54 length/GM) and a moderately broad (0.69 width/GM) palate among the frugivorous lineage. We observed all major forms of palate shape (short, long, and intermediate^50^); in phyllostomids. Primates also presented variation in craniofacial morphology (0.60–0.94 length/GM; 0.42–0.52 width/GM. Overall, among primates, *Papio anubis* presented the more elongated face (0.94 palate length/GM, 0.47 width/GM).

Similarly, great variation in RUNX2 Q/A repeats and flanking control regions was evident both in phyllostomid bats and primates (Table 1). Overall, phyllostomid bats presented greater variation in number of glutamate and alanine residues than did primate species (Fig. 1C). Frugivorous phyllostomids presented the widest range, with a Q/A ratio varying from 1.21 to 2.33 (Table 1 and Fig. 1C).

**Table 1:** Species examined in this study with craniofacial measurements and RUNX2 Q/A ratios. GM: geometric mean.

Group	Feeding habit	Species	Palate (mm)/GM			RUNX2		
			Length/GM	Width/GM	Zygomatic/GM	Q	A	Q/A Ratio
*New World leaf-nosed bats*	*Carnivory*	*Chrotopterus auritus*	*0.71*	*0.62*	*1.10*	23	17	*1.35*
	*Frugivory*	*Artibeus glaucus*	*0.64*	*0.75*	*1.10*	20	10	*2.00*
		*Artibeus gnomus*	*0.60*	*0.78*	*1.11*	21	10	*2.1*
		*Artibeus jamaicensis*	*0.70*	*0.76*	*1.11*	23	13	*1.77*
		*Artibeus lituratus*	*0.67*	*0.80*	*1.10*	23	10	*2.3*
		*Artibeus obscurus*	*0.68*	*0.77*	*1.12*	23	13	*1.77*
		*Artibeus watsoni*	*0.63*	*0.75*	*1.06*	21	9	*2.33*
		*Carollia perspicillata*	*0.73*	*0.63*	*1.07*	19	15	*1.27*
		*Carollia subrupha*	*0.74*	*0.63*	*1.04*	18	14	*1.29*
		*Chiroderma doriae*	*0.72*	*0.79*	*1.12*	26	13	*2.00*
		*Chiroderma villosum*	*0.74*	*0.80*	*1.11*	23	12	*1.92*
		*Rhinophylla fisherae*	*0.69*	*0.67*	*1.09*	20	14	*1.43*
		*Rhinophylla pumilio*	*0.68*	*0.67*	*1.07*	17	13	*1.31*
		*Sturnira lilium*	*0.63*	*0.67*	*1.18*	20	12	*1.67*
		*Sturnira tildae*	*0.66*	*0.68*	*1.21*	17	14	*1.21*
		*Vampyressa pusilla*	*0.67*	*0.78*	*1.11*	23	13	*1.77*
		*Vampyrodes caraccioli*	*0.71*	*0.82*	*1.14*	21	13	*1.62*
	*Strict frugivory*	*Pygoderma bilabiatum*	*0.54*	*0.69*	*1.27*	22	11	*2.00*
	*Hematofagy*	*Desmodus rotundus*	*0.44*	*0.60*	*1.24*	18	12	*1.5*
		*Diphylla ecaudata*	*0.51*	*0.62*	*1.23*	19	13	*1.46*
	*Insectivory*	*Glyphonicteris sylvestris*	*0.81*	*0.64*	*1.03*	20	15	*1.33*
	*Nectarivory*	*Anoura caudifer*	*0.90*	*0.51*	*0.99*	21	14	*1.5*
		*Anoura geoffroyi*	*0.91*	*0.52*	*0.99*	19	14	*1.36*
		*Glossophaga commissarisi*	*0.85*	*0.54*	*0.99*	21	15	*1.4*
		*Glossophaga soricina*	*0.86*	*0.54*	*0.99*	20	14	*1.43*
		*Lonchophylla thomasi*	*0.83*	*0.54*	*1.00*	18	15	*1.2*
	*Omnivory*	*Lophostoma silviculum*	*0.77*	*0.60*	*1.04*	19	15	*1.27*
		*Mimon benettii*	*0.75*	*0.66*	*1.07*	17	13	*1.31*
		*Gardnerycteris crenulatum*	*0.72*	*0.69*	*1.01*	17	15	*1.13*
		*Phylostomus elongatus*	*0.75*	*0.70*	*1.09*	19	15	*1.27*
		*Phylostomus hastatus*	*0.74*	*0.63*	*1.09*	19	14	*1.36*
*Primates*	*NWM*	*Leontopithecus rosalia*	*0.77*	*0.52*	*1.26*	23	17	*1.35*
		*Sapajus robustus*	*0.80*	*0.42*	*1.40*	21	16	*1.31*
		*Brachytheles arachnoides*	*0.80*	*0.42*	*1.34*	21	16	*1.31*
		*Callithrix hummelifera*	*0.72*	*0.51*	*1.32*	21	17	*1.24*
		*Callithrix pygmaea*	*0.72*	*0.47*	*1.26*	19	16	*1.19*
		*Callithrix geoffroyi*	*0.74*	*0.49*	*1.29*	21	17	*1.24*
	*OWM*	*Pan paniscus* ^*a*^	*0.73*	*0.51*	*1.33*	16	17	*0.94*
		*Pan troglodites* ^*b*^	*0.75*	*0.51*	*1.29*	25	17	*1.47*
		*Macaca fasciculari* ^*a*^	*0.88*	*0.49*	*1.32*	24	17	*1.41*
		*Gorilla gorilla* ^*b*^	*0.75*	*0.46*	*1.25*	22	17	*1.29*
		*Homo sapiens* ^*b*^	*0.60*	*0.47*	*1.26*	23	17	*1.35*
		*Homo neanderthalensis* ^c*^	*0.63*	*0.49*	*1.38*	23	17	*1.35*
		*Papio anubis* ^*b*^	*0.94*	*0.47*	*1.23*	24	17	*1.41*

^a^NCBI, ^b^ENSEMBL, ^c^UCSC,

*http://neandertal.ensemblgenomes.org/Homo_sapiens/Transcript/Summary?db=core;t=ENST00000359524.

### Correlation between Q/A ratios and palate length and width

**Figure 2:**
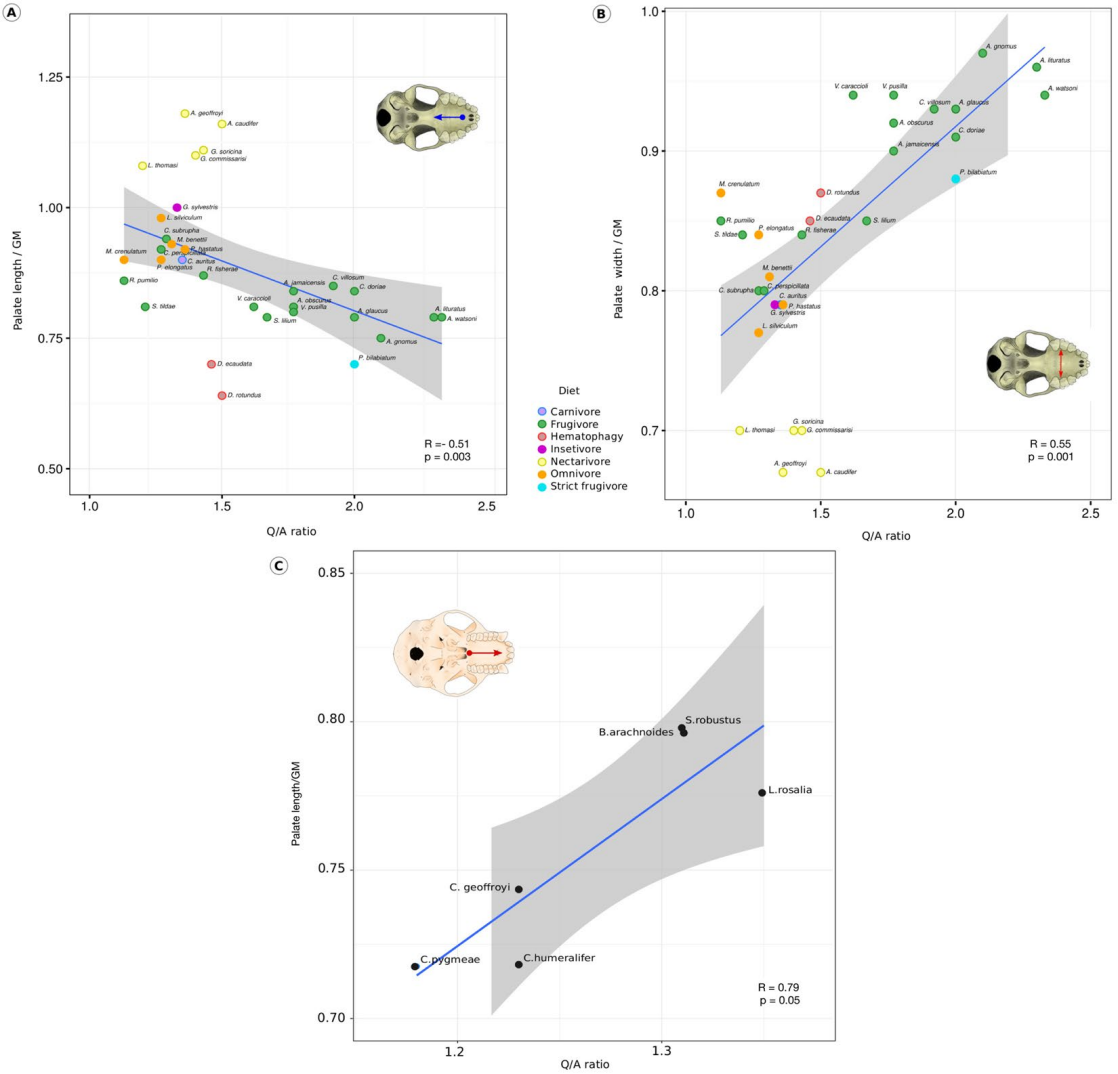
Correlations between Q/A ratios and palate lengths (**A**), phyllostomids; (**C**) New World monkeys) and width (**B**), phyllostomids).

Spearman’s correlation test for the phyllostomid bats data showed a significant negative correlation between Q/A ratio and palate length r = −0.51 (p = 0.01) and significant positive correlation between palate width and Q/A ratio, r = 0.55 (p < 0.01) (Fig. 2A,B). Spearman’s correlation test for the primates data (NWM and OWM analysed together), and for OWM (analysed separately) showed no significant association between the Q/A ratio and craniofacial morphological traits (NWM + OWM: palate length × Q/A ratio: r = 0.44, p = 0.12; palate width × Q/A ratio: r = 0.07, p = 0.80; OWM: palate length × Q/A ratio: r = 0.58, p = 0.17; palate width × Q/A ratio: r = 0.29, p = 0.52). For NMW (analysed separately), we found a positive marginally significant association between palate length and Q/A ratio (r = 0.79, p = 0.05) (Fig. 2C).

Bayesian PCM was also used to estimate the correlation coefficient between palate and Q/A ratios in both groups, corrected for phylogenetic relatedness, evolutionary rates and divergence times. In accordance with the previous findings, these analyses showed a positive correlation between Q/A ratio and palate width (r = 0.2910; pp* = *0.94), and a negative correlation between Q/A ratio and palate length (r = −0.2260; pp = 0.13) for phyllostomid bats (Table 2). The craniofacial variation of primates in general did not correlate with Q/A ratios. However, when the dataset is split in two subgroups (NWM and OWM), we found a significant correlation between palate length and Q/A ratios for NWM (r = 0.583, pp = 0.93)

**Table 2:** Bayesian coefficient of correlation (estimated with CoEvol program, see material and methods section for details) between craniofacial measures (PL, palate length; PW, palate width; ZW, zygomatic width) and Q/A ratio.

	PL	*pp*	PW	*pp*	ZW	*pp*
Phyllostomids vs. Q/A ratio	−0.226	0.13*	0.291	0.94*	0.004	0.51 ^ns^
Primates vs. Q/A ratio	0.095	0.63 ^ns^	−0.017	0.47 ^ns^	−0.116	0.34 ^ns^
NWM vs. Q/A ratio	0.583	0.93*	0.237	0.71 ^ns^	0.275	0.73 ^ns^
OWM vs. Q/A ratio	0.051	0.56 ^ns^	−0.087	0.40 ^ns^	−0.176	0.30 ^ns^

Statistical significance is given by posterior probability (pp). *Statistically significant; ^ns^ Not significant.

For phyllostomids, the PGLS results showed a phylogenetic signal (Pagel’s lambda = 1) associated with palate length and width, and with zygomatic width (Table 3). The regression between phenotypic traits and Q/A ratios showed that palate width is positively associated with *RUNX2* variation (adjusted R² = 0.19, p = 0.01). For primates (NWM and OWM analysed together) the PGLS results showed a phylogenetic signal associated with palate length and width. In that case, we found no significant association between phenotypic traits and Q/A ratios (Table 3). For NMW (analysed separately), the PGLS results presented phylogenetic signal associated with palate length and width, and a positive marginally significant association between palate length and Q/A ratio (adjusted R² = 0.55, p = 0.06) (Table 3). Finally, in the OWM, we found phylogenetic signal associated with palate length and no association between phenotypic traits and Q/A ratios in the PGLS analyses (Table 3).

**Table 3:** Results of phylogenetic generalized least squares (PGLS) models exploring the putative association between RUNX2 Q/A ratios and relative palate-length and width in phyllostomid bats and primates, depicting the magnitude of phylogenetic signal (Pagel’s lambda).

Group	Model	r^2^	Estimate	p	Pagel’s lambda
Phyllostomid bats	Q/A ratio x PL	0.06	−0.09	0.09	1000
	Q/A ratio x PW	0.19	0.08	0.01	1000
	Q/A ratio x ZW	−0.03	0.02	0.72	1000
Primates	Q/A ratio x PL	−0.07	0.04	0.63	994
	Q/A ratio x PW	−0.09	−0.01	0.90	933
	Q/A ratio x ZW	−0.06	−0.06	0.59	0
Only NWM	Q/A ratio x PL	0.55	0.27	0.06	1000
	Q/A ratio x PW	−0.18	0.32	0.66	1000
	Q/A ratio x ZW	−0.06	0.31	0.44	0
Only OWM	Q/A ratio x PL	−0.18	0.04	0.77	1000
	Q/A ratio x PW	−0.11	−0.03	0.55	0
	Q/A ratio x ZW	−0.09	−0.09	0.51	0

### Correlation between Q/A ratios and morphological integration index

**Figure 3:**
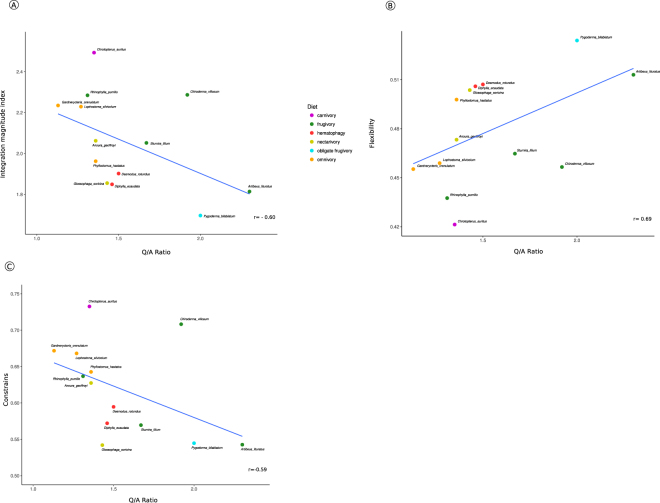
(**A**) Plot of the overall integration magnitude (ICV) and Q/A ratio for phyllostomid bats. (**B**) Plot of the evolutionary flexibility index and Q/A ratio for phyllostomid bats. (**C**) Plot of the evolutionary constraints index and Q/A ratio for phyllostomid bats.

The morphological integration index (ICV), obtained for phyllostomid bats, exhibited strong negative correlation with Q/A ratios (r = −0.6, p = 0.02; Fig. 3A). Additionally, the evolutionary flexibility index was strongly correlated with Q/A ratios (r = 0.7, p < 0.01; Fig. 3B), and the constraints index exhibited negative correlation with Q/A ratios (r = −0.6, p = 0.03; Fig. 3C). Spearman’s correlation test for the primates data analysed together and separately showed no significant association between the Q/A ratio and ICV, flexibility and constraints indexes. The lack of correlation observed for primates data might be due to the relatively small sample sizes for both NWM and OWM clades.

## Discussion

### Frugivorous phyllostomid bats: a hotspot of variation

The Phyllostomidae family includes subfamilies that are recognised mainly by their feeding habits and associated morphological adaptations^27,28,51^. Phytophagous phyllostomids (frugivorous and nectar feeding species) evolved in nested clades among ancestral animalivorous species, and previous studies have proposed that the insectivorous ancestor of all phyllostomids also fed on plants^28,31,51,52^. In the present study, the largest variation in the Q/A ratios was found in frugivorous phyllostomids (Q/A ratio varying from 1.21 to 2.33; see Table 1). The feeding specialisation in the subfamily Stenodermatinae varies from primary frugivory (feed on fruits and complement diet with other items, e.g. *Artibeus*, *Carollia*, *Sturnira*, *Vampyrodes*, *Vampyressa*) to obligate frugivory (feed on hard fruits, e.g. *Pygoderma bilabiatum*). The origin of the Stenodermatinae clade is associated with a significant increase in the species diversification rate and a high bite performance skull^27,53^. The anatomical form of the palate in frugivorous phyllostomids (broad structure^50^) likely favoured a mechanical advantage for this group. When frugivorous vertebrates emerged ca. 25–38 Myr ago, among all other lineages of bats, only phyllostomids fed on fruits^31^. Thus, they explored a new adaptive zone that likely required remarkable cranial and dental adaptations. Sorensen *et al*.^50^ suggested that the diverse palate shapes of phyllostomids are the result of subtle evolutionary changes in later developmental events.

### Magnitude of skull integration in bats and primates: flexibility to evolve

In contrast to the remarkable stability observed in the patterns of covariation in the mammalian skull^54–57^ the magnitudes of inter-trait association, estimated by the average squared phenotypic correlation between traits (*r*^*2*^), are highly variable^56^. For the same cranial measurements, primates (chimpanzees, humans, and gorillas) and Chiroptera (*Molossus molossus*, *Myotis nigricans*, and *Artibeus lituratus* - Family Molossidae, Vespertilionidae, and Phyllostomidae, respectively) have *r*^*2*^ values under 0.10, whereas Carnivora (*Cerdocyon thous, Leopardus*, *Eira, Nasua* - Family Canidae, Felidae, Mustelidae, and Procyonidae, respectively) display values on the order of 0.12–0.20, and marsupials have *r*^*2*^ values of 0.30–0.50^56,58^.

The high variability in the magnitude of inter-trait correlations has potential important consequences for the evolution of these clades^59^. Higher magnitudes of skull integration are associated with constrained evolutionary responses to selection, which would reduce adaptive flexibility to evolve in directions other than those driven by the tight inter-trait associations, at least on short-term^46,59,60^. Alternatively, lower magnitudes indicate that most traits are weakly connected and that there is more potential to evolve across several axes of variation. Species with lower magnitudes of trait association could evolve in more directions, therefore presenting less potential evolutionary constraint and greater evolutionary flexibility^59^. Thus, the different integration magnitudes in phyllostomid bats in relation to the *RUNX2* gene (Fig. 3A–C) together with the probable differences in the reaction norms of this gene within this clade, could explain the differences in the genotype–phenotype relationships observed, as well as the contrasting patterns described for other lineages of mammals.

The mice genome informatics database (http://www.informatics.jax.org/vocab/mp_ontology/MP:0020039) lists several known skeleton phenotypes compiled so far and associated with *RUNX2* mutants. In this context, we could assume that delayed or increased bone ossification (both endochondral and intramembranous) might explain the opposed association of *RUNX2* variation and/or integration and facial forms we observe in different groups of mammals. By affecting bone ossification the locus potentially can impact allometric size variation within population, which in turn would usually impact mostly facial traits due to its late development (see^61,62^ for craniofacial development in mammals). Consequently it would lead to an increase or decrease in correlation among facial traits and thus to a larger or smaller morphological integration. Thus, the strong positive correlation found between evolutionary flexibility index and the *RUNX2* gene (Fig. 3B) suggests a potential association between the high potential ability for craniofacial traits evolution in phyllostomid bats and *RUNX2* variation. The increased ability to promote bone tissues formation might have played a fundamental role during transitions to feeding specializations, thus facilitating evolutionary change. Finally, since the ratio of glutamine to alanine residues in the RUNX2 protein might influence the regulation of bone development, our results indicate that Q/A ratio can act as a key feature for natural selection to operate in these species.

### Long, short, or broad faces: what is the impact of RUNX2?

Our results clearly showed contrasting patterns of association between tandem repeats of *RUNX2* Q/A and palate shape in the highly diverse groups of phyllostomid bats and primates (Tables 1, 2 and Fig. 2). Until recently, studies on marsupials and placental mammals, including domestic dog breeds, evidenced two contrasting patterns: i) a correlation of Q/A ratios and face lengths^10^; or ii) a non-association of these traits^11,12^. Sears *et al*.^10^ suggested a pattern of increase in face length of carnivores due to a higher Q/A ratio. In contrast, the results obtained in our study point to a reduction in length and a widening of the maxilla, which would lead to shorter and broader faces in phyllostomid bats. However, in NWM, the result is consistent with that described for the order Carnivora. Overall, our results support the main idea proposed by Sears *et al*.^10^, reinforcing the role of the Q/A ratio as a flexible genetic mechanism that would rapidly change the time of ossification of the skull throughout development.

In this context, the findings of the present study propose a complex scenario, where the permissiveness of this genetic adjustment system created by the variability in the Q/A ratios of the *RUNX2* gene is indirect, and does not lead to a specific phenotype. However, throughout the history of a lineage (at the species or family level), *RUNX2* variability acts with external factors and evolutionary constraints, together with other genes, as a facilitator for the adaptive morphological changes currently found. In the case of phyllostomids, these changes seem to have been selected by dietary constraints, corroborating the phenotype–environment correlation^27,32,33,41,63^. Overall, it should be noted that many genetic, epigenetic, and environmental attributes modulates the expression of genetic variance and interact with developmental networks to produce the available phenotypic variation. Therefore, the results of the present study seem to agree with Alberch’s^64^ view on the conceptualisation of genotype-phenotype association, i.e. genes do not directly specify the development or the shape of an organism; they are only one of the several causal factors that jointly determine the phenotype. Thus, developmental events are able to ambiguously affecting, or be affected, by gene expression.

## Material and Methods

### *RUNX2* sequence data

Samples from 31 phyllostomid bat species from a wide range of feeding habits and craniofacial morphologies were surveyed in this study (electronic supplementary material, Table S1). Tissues and skulls individually paired are deposited in the Department of Zoology collection at the Fundação Universidade Regional de Blumenau (FURB). Additionally, six New World primate species (*Leontopithecus rosalia*, *Spajus robustus*, *Brachytheles arachnoides*, *Callithrix hummelifera*, *Callithrix pygmaea*, *Callithrix geoffroyi*), from the Laboratório de Evolução Molecular of the Departamento de Genética, Universidade Federal do Rio Grande do Sul, were used. Five species of Catarrhini (*Pan paniscus*, *Papio anubis*, *Gorilla gorilla*, *Pan troglodytes*, *and Macaca fascicularis*) were considered using the ENSEMBL (www.ensembl.org, release 86, [A. Yates]) and NCBI (http://www.ncbi.nlm.nih.gov/genbank/[Benson]) genomic databases.

Additionally, UCSC (https://genome.ucsc.edu/index.html) and Neandertal Ensembl (http://neandertal.ensemblgenomes.org/data_info.htm) genomes were accessed to obtain modern human and Neandertal data. The DNeasy Blood & Tissue Kit (Qiagen, Germantown, MD, USA) was used to extract and isolate genomic DNA from muscle samples for the 37 species. The polymerase chain reaction (PCR) was carried out to amplify the target *RUNX2* exon, using primers and conditions described by Pointer *et al*.^11^, standardising the PCR conditions for the different species surveyed. Shrimp alkaline phosphatase and exonuclease I enzymes (GE Healthcare, Chicago, IL, USA) were used to purify the PCR products. Amplicons were sequenced in an ABI3730xl (Applied Biosystems Inc., Foster City, CA, USA) sequencer, using the same primers employed in the PCR for both DNA strands. Sequences were analysed using the ClustalW algorithm, implemented in the Codon Code Aligner software (CodonCode Corporation, Dedham, MA, USA)^65^. A prior screening test was performed, which considered only the sequences presenting conserved regions flanking the Q/A motif. These regions were then used as reference markers for the Q/A motif. The Q/A ratio for each specimen was calculated by dividing the number of repeated glutamine residues by the number of repeated alanine residues in tandem^10,11^ (Table 1).

### Samples and linear measurements

**Figure 4:**
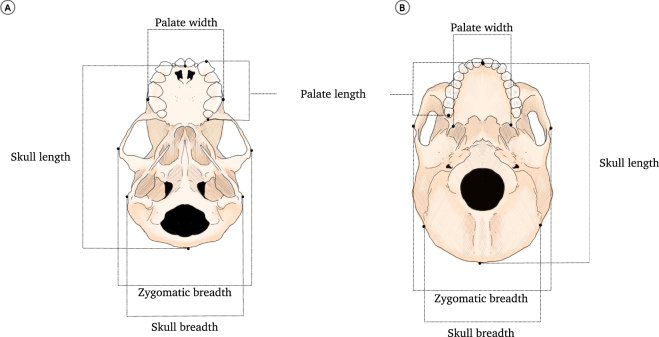
Craniofacial measurements recorded in this study. (**A**) Ventral view of the phyllostomid bat skull. (**B**) Ventral view of a primate skull. See also the electronic supplementary material, Table S2, for detailed descriptions of the measurements used in this study.

We measured 31 phyllostomid skulls (the same specimens sequenced for RUNX2) using a digital calliper, and obtained the primate data set employing a combination of craniometric data recorded by Marroig and Cheverud^66^ and Oliveira *et al*.^54^ using a Microscribe 3DMX. From these data, we obtained five linear measurements from the ventral crania of all specimens: palate length, palate width, skull length, skull width, and zygomatic breadth (Fig. 4, see also the electronic supplementary material, Table S2, for detailed measurement descriptions). These linear measurements were chosen to reflect variations in skull form among species and to represent the dimensions of palate width and length both in phyllostomid bats and primates^54,63,66,67^.

Additionally, evolutionary alterations in palate morphology are thought to underlie much of the feeding specialisations in bats^50^. We calculated the geometric mean for all measurements and standardised the palate length and width for each specimen by dividing each measurement by the geometric mean (GM) (Table 1). Only skulls from adult specimens were measured. For both primates and bats, specimens were considered adults when the basisphenoid and basioccipital joint was completely fused.

### Statistical analysis

In an initial set of analysis, we performed Spearman’s correlation test to investigate the association between *RUNX2* tandem repeat ratios and palate length and width. Additionally to this traditional statistical method, a Bayesian phylogenetic comparative method (PCM) approach was implemented in CoEvol 1.4 software^68^ to investigate the correlation between *RUNX2* Q/A ratio and palate length and width, taking into account phylogenetic relationships, evolutionary rates, and divergence time under a Brownian motion model of evolution. In the CoEvol results, a posterior probability (pp) close to 1 represents a strong statistical support for a positive correlation, and a pp close to 0 indicates a supported negative correlation. Two data sub-sets for each taxonomic group (bats and primates) were analysed: I, morphometric measures for each species, corrected by geometric mean of all measures and II, phylogenetic relationships estimated from neutral markers (i.e., mitochondrial genes obtained from public databases). We ran CoEvol for 30,000 MCMC (total number of cycles) and considered the analysis finished when all effective sample size values reached at least 300^68^.

Finally, we performed a phylogenetic generalised linear model (PGLS) using the R function (pgls) of the “caper” R package^69^ to investigate the magnitude of phylogenetic signal associated with phenotypic (palate length and width - dependent variables) and genotypic (Q/A region - independent variable) changes. This function fits a linear model controlling for phylogenetic non-independence between data. We also used this parameter for comparative analyses with the results of previous studies on carnivores^10^ and other placental mammals^11^. We assume that all the three analytical methods employed in our study are integrative and represents different levels of complexity, ranging from a more general exploratory analysis (Spearman’s correlation) to a more complex phylogenetic comparative methods (PGLS and Bayesian).

### Q/A ratios and morphological integration index

The magnitudes of integration among traits were obtained by using the coefficient of variation of the eigenvalues (ICV^70^;). This index was calculated for primates^56,59^ and phyllostomids (Rossoni et al. *manuscript in preparation*) as the standard deviation of the eigenvalues (σ_(λ)_) divided by the average of the eigenvalues (λ). The flexibility and constraints index were also provided from the author’s cited above. The evolutionary flexibility index captures the ability of a species to respond in direction of selection, while the evolutionary constraints index captures the relative influence of the line of least resistance (first principal component) on the direction of the evolutionary responses^59^. These measurements are presented in the electronic supplementary material, Table S3 (see also^58,59,70^ for a detailed description of these index estimates). We performed Spearman’s correlation test in order to investigate the association between RUNX2 tandem repeat ratios and ICV, flexibility and constraints index.

## Electronic supplementary material

Supplementary Material
