# Supplementary material for: Contrasting patterns of RUNX2 repeat variations are associated with palate shape in phyllostomid bats and New World primates
Source: Sci Rep. 2018 May 18;8:7867. doi: 10.1038/s41598-018-26225-7 (PMC5959863; doi:10.1038/s41598-018-26225-7)
Supplement: Supplementary file 1 — Supplementary Material [file 41598_2018_26225_MOESM1_ESM.pdf]

Supplementary Material

Contrasting patterns of RUNX2 repeat variations are associated with palate shape in phyllostomid bats and New World primates

Tiago Ferraz[1, 2], Daniela M. Rossoni[2], Sérgio. L. Althoff[3], Alcides. Pissinatti[4], Vanessa R. Paixão-Cortês[5], Maria Cátira Bortolini[1], Rolando González-José[6], Gabriel Marroig[2], Francisco M. Salzano[1], Gislene L. Gonçalves[1, 7] and Tábita Hünemeier[2*]

[1]Department of Genetics, Biosciences Institute, Federal University of Rio Grande do Sul, 91501-970 Porto Alegre, RS, Brazil

[2]Department of Genetics and Evolutionary Biology, Biosciences Institute, University of São Paulo, 05508-900 São Paulo, SP, Brazil

[3]Regional University Foundation, Blumenau, SC, Brazil

[4]Biology Department, Federal University of Bahia, Salvador, BA, Brazil

[5]Rio de Janeiro Primatology Center, 20940-200 Rio de Janeiro, RJ, Brazil;

[6]Patagonian Institute of Social and Human Sciences, National Council for Scientific and Technological Research-CONICET, U9120ACD, Puerto Madryn, Argentina;

[7]Department of Environmental Resources, Faculty of Agrarian Sciences, University of Tarapacá, Arica, Chile

***Author for correspondence:** T. Hünemeier, hunemeier@usp.br. Tel: +5511 (3391-8759).

Running head: RUNX2 and palate shape evolution in bats and primates

Table S1. Species examined in this study with voucher's ID, craniofacial measurements and geometric mean.

Order	Feeding habit	Species	Voucher seq ID's	Voucher ID's	Palate (mm)			Geometric mean
					Length	Width	Zigomatic	
Leaf-nosed bats	Carnivorous	*Chrotopterus auritus*	*SLA 3509*	*SLA 3509*	***13.00***	***11.50***	***20.30***	***18.42***
	Frugivorous	*Artibeus glaucus*	*SLA 4047*	*SLA 4047*	***6.50***	***7.60***	***11.20***	***10.19***
		Artibeus gnomus	*SLA 4062*	*SLA 4062*	***6.00***	***7.80***	***11.10***	***10.00***
		Artibeus jamaicensis	*SLA 3172*	*SLA 3172*	***11.40***	***12.30***	***18.00***	***16.24***
		Artibeus lituratus	*SLA 2236*	*SLA 2236*	***12.30***	***14.80***	***20.40***	***18.47***
		Artibeus obscurus	*SLA 4068*	*SLA 4068*	***10.10***	***11.50***	***16.80***	***14.96***
		Artibeus watsoni	*SLA4059*	*SLA4059*	***7.00***	***8.30***	***11.80***	***11.14***
		Carollia perspicillata	*SLA 3808*	*SLA 3808*	***8.00***	***7.00***	***11.80***	***11.03***
		Carollia subrupha	*SLA 4039*	*SLA 4039*	***8.00***	***6.80***	***11.30***	***10.85***
		Chiroderma doriae	*SLA 3795*	*SLA 3795*	***12.10***	***13.20***	***18.80***	***16.77***
		Chiroderma villosum	*SLA 3120*	*SLA 3120*	***10.70***	***11.70***	***16.10***	***14.54***
		Rhinophylla fisherae	*SLA 3457*	*SLA 3457*	***6.50***	***6.30***	***10.30***	***9.45***
		Rhinophylla pumilio	*SLA 3039*	*SLA 3039*	***6.30***	***6.20***	***10.00***	***9.32***
		Sturnira lilium	*SLA 2538*	*SLA 2538*	***7.70***	***8.20***	***14.40***	***12.20***
		Sturnira tildae	*SLA 3514*	*SLA 3514*	***8.70***	***9.00***	***15.90***	***13.19***
		Vampyressa pusilla	*SLA 2900*	*SLA 2900*	***7.50***	***8.80***	***12.40***	***11.22***
		Vampyrodes caraccioli	*SLA 3481*	*SLA 3481*	***11.90***	***13.80***	***19.20***	***16.87***
	strict frugivorous	*Pygoderma bilabiatum*	*SLA 3759*	*SLA 3759*	***5.90***	***7.50***	***13.80***	***10.87***
	Hematofagous	*Desmodus rotundus*	*SLA 2718*	*SLA 2718*	***4.50***	***6.10***	***12.70***	***10.25***
		Diphylla ecaudata	*SLA 3321*	*SLA 3321*	***5.30***	***6.50***	***12.80***	***10.44***
	Insectivorous	*Glyphonicteris sylvestris*	*SLA 3534*	*SLA 3534*	***8.50***	***6.70***	***10.80***	***10.52***
	Netarivorous	*Anoura caudifer*	*SLA 2054*	*SLA 2054*	***8.70***	***5.00***	***9.60***	***9.72***
		Anoura geoffroyi	*SLA 3178*	*SLA 3178*	***10.40***	***5.90***	***11.2***	***11.37***
		Glossophaga commissarisi	*SLA 3181*	*SLA 3181*	***8.00***	***5.10***	***9.40***	***9.45***
		Glossophaga soricina	*SLA 2688*	*SLA 2688*	***8.10***	***5.10***	***9.30***	***9.44***
		Lonchophylla thomasi	*SLA 3465*	*SLA 3465*	***7.70***	***5.00***	***9.30***	***9.29***
	Omnivoro us	*Lophostoma silviculum*	*SLA2884*	*SLA2884*	***9.80***	***7.70***	***13.30***	***12.81***
		Mimon benettii	*SLA 2823*	*SLA 2823*	***9.80***	***8.60***	***14.00***	***13.07***
		Gardnerycteris	*SLA 4147*	*SLA 4147*	***8.30***	***8.00***	***11.70***	***11.57***

			crenulatum						
			Phylostomus elongatus	*SLA 4049*	*SLA 4049*	***11.60***	***10.80***	***16.90***	***15.47***
			Phylostomus hastatus	*SLA3130*	*SLA3130*	***13.60***	***11.70***	***20.10***	***18.50***
Primates	***NWM***	***Frugivorous***	*Leontopithecus rosalia*	*P 2256*	**	***21.19***	***14.34***	***34.45***	***27.35***
		Folivorous	*Sapajus robustus*	*P 2132*	**	***35.13***	***18.67***	***61.80***	***44.0***
			Brachytheles arachnoides	*P 2160*	**	***42.52***	***22.54***	***71.33***	***53.33***
		Granivorous	*Callithrix hummelifera*	*P 1855*	**	***15.88***	***11.19***	***29.23***	***22.11***
			Callithrix pygmaea	*P 2074*	**	***11.96***	***7.85***	***21.02***	***16.66***
			Callithrix geoffroyi	*P 2175*	**	***16.67***	***10.96***	***29.00***	***22.42***
	OWM	***Frugivorous***	*Pan paniscus* [a]	*XM_003833214*	**	***60.20***	***42.24***	***110.64***	***82.99***
			Pan troglodites [b]	*XM_001142454*	**	***71.68***	***48.68***	***122.66***	***95.43***
			Macaca fascicularis [a]	*XM_005552840*	**	***48.82***	***27.40***	***73.57***	***55.69***
		Folivorous	*Gorilla gorilla* [b]	*M_004044127*	**	***99.05***	***60.11***	***165.55***	***132.02***
		Omnivorous	*Homo sapiens* [b]	*XM_006715231*	**	***56.37***	***44.00***	***119.08***	***94.27***
			Homo neanderthalensis [c*]	*ENSG00000124813*	**	***69.5***	***53.57***	***151.98***	***110.38***
			Papio anubis [b]	*XM_003897680*	**	***89.20***	***44.17***	***117.09***	***94.91***

[a,] NCBI , [b] ENSEMBL, [c] UCSC, * http://neandertal.ensemblgenomes.org/Homo_sapiens/Transcript/Summary?db=core;t=ENST00000359524

*** Marroig and Cheverud (2001) and Oliveira et al.(2009)*

Table S2. Detailed descriptions of the craniofacial measurements used in this study. See also Figure 3 in the manuscript for measurements illustration in primates and chiropteran skull.

	Abbreviation	Description
Leaf-nosed bats	Skull length	Distance from the posteriormost point on the occiput to the anteriormost point on the premaxilla
	Skull breadth	Greatest breadth across the mastoid region
	Zygomatic breadth	Greatest breadth across the zygomatic arches
	Palate length	Distance from the anteriormost surface of the upper canine to the posteriormost surface of the crown of M3
	Palate width	Greatest width of palate across labial margins of the alveoli of M2s
Primates	Skull length	Distance from interincisive suture to the midsagittal lambdoidea suture
	Skull breadth	Distance across the left and right occipitoparietal suture
	Zygomatic breadth	Distance across the left and right temporozygomatic suture
	Palate length	Distance from interincisive suture to the palatomaxillary suture
	Palate width	Distance across the left and right palatomaxillary suture

Table S3. The RUNX2 tandem repeat ratios, overall integration magnitude (ICV) and the evolutionary flexibility and constraints index.

	Species	Q/A Ratio	ICV	flexibility	constraints
Leaf-nosed bats	*Artibeus_lituratus*	2.30	1.81	0.51	0.54
	Chrotopterus_auritus	1.35	2.49	0.42	0.73
	Chiroderma_villosum	1.92	2.29	0.46	0.71
	Rhinophylla_pumilio	1.31	2.28	0.44	0.64
	Sturnira_lilium	1.67	2.05	0.46	0.57
	Pygoderma_bilabiatum	2.00	1.70	0.53	0.54
	Desmodus_rotundus	1.50	1.90	0.51	0.59
	Diphylla_ecaudata	1.46	1.85	0.51	0.57
	Anoura_geoffroyi	1.36	2.06	0.47	0.63
	Glossophaga_soricina	1.43	1.86	0.50	0.54
	Lophostoma_silvicolum	1.27	2.23	0.46	0.67
	Gardnerycteris_crenulatum	1.13	2.24	0.46	0.67
	Phyllostomus_hastatus	1.36	1.96	0.50	0.64
Primates	*Leontopithecus rosalia*	1.35	2.60	0.40	0.45
	Sapajus robustus	1.31	3.19	0.35	0.72
	Brachytheles arachnoides	1.31	1.77	0.51	0.41
	Callithrix hummelifera	1.24	1.75	0.54	0.50
	Callithrix pygmaea	1.19	1.78	0.53	0.49
	Callithrix geoffroyi	1.24	2.14	0.46	0.55
	Pan paniscus	0.94	1.87	0.51	0.30
	Pan troglodites	1.47	1.88	0.53	0.50
	Macaca fascicularis	1.41	3.70	0.34	0.77
	Gorilla gorilla	1.29	2.23	0.48	0.55
	Homo sapiens	1.35	1.76	0.54	0.53
	Papio anubis	1.41	4.22	0.29	0.66
